# Zinc-Doped Calcium Phosphate Nanoagonists Amplifies cGAS-STING Signaling for Boosting Pyroptosis-Induced Cancer Immunotherapy

**DOI:** 10.3390/jfb17060308

**Published:** 2026-06-22

**Authors:** Bangliu Yang, Xinyu Li, Mingyue Zhang, Shiyao Guo, Xueqian Wang, Peiran Chen, Dongqin Yu, Chao Qi, Kaiyong Cai

**Affiliations:** Key Laboratory of Biorheological Science and Technology, Ministry of Education, College of Bioengineering, Chongqing University, Chongqing 400044, China

**Keywords:** calcium phosphate, drug delivery, pyroptosis, cGAS-STING, immunotherapy

## Abstract

The combination of chemotherapy and immunotherapy represents a promising approach that leverages their complementary benefits. However, the side effects resulting from off-target effects and the low efficiency of immune activation remain a significant concern. Herein, we developed a zinc-doped calcium phosphate (ZCP) nanocarrier for the delivery of the chemotherapeutic drug doxorubicin (DOX). By further encapsulating whole proteins from 4T1 breast cancer cells, we constructed a novel nanodrug delivery system named ZCPDM. This system enables specific targeting of tumor cells and undergoes intracellular degradation to release DOX, Zn^2+^, and Ca^2+^. As a chemotherapeutic agent, DOX induces apoptosis while significantly elevating intracellular reactive oxygen species (ROS), thereby enhancing cytotoxicity. This leads to DNA damage and the release of chromosomal fragments. These DNA fragments, together with Zn^2+^, activate the cGAS-STING signaling pathway and trigger pyroptosis, which promotes more efficient recognition and clearance of tumor cells by the immune system. Through these dual mechanisms, ZCPDM effectively combines chemotherapy and immunotherapy. The anti-tumor efficacy and underlying mechanisms were validated at the cellular level. Furthermore, studies in tumor-bearing mice demonstrated its robust anti-tumor performance and ability to suppress tumor recurrence, along with good biosafety. This targeted drug delivery system achieves safe and synergistic chemo-immunotherapy through homologous targeting-mediated pyroptosis and activation of the cGAS-STING pathway, offering a novel and promising strategy for cancer treatment.

## 1. Introduction

Cancer remains one of the leading causes of death worldwide [1]. Conventional clinical treatments include surgery, chemotherapy, and radiotherapy [2]. Among these, chemotherapy employs cytotoxic drugs to inhibit tumor progression and prolong patient survival [3]. However, the clinical utility of chemotherapy is limited by several drawbacks, such as poor solubility and stability of chemotherapeutic agents, severe side effects, and low bioavailability [4,5]. These limitations contribute to off-target effects, tumor drug resistance, and immediate or long-term severe toxicities [6]. As a result, chemotherapy alone often leads to suboptimal clinical outcomes [7]. The combination of chemotherapy and immunotherapy represents a highly promising approach in oncology [8,9,10,11]. It leverages chemotherapeutic agents to enhance tumor immunogenicity, while immunotherapy effectively eliminates residual tumor cells, resulting in a synergistic anti-tumor effect [12,13,14]. However, immunogenic cell death (ICD)-based therapy is largely limited by low tumor immunogenicity and the immunosuppressive tumor microenvironment (ITM) [15].

In recent years, nanotechnology-based drug delivery systems have opened up new possibilities for the precise implementation of such combination strategies [16,17,18]. The nanoscale size of nanocarriers facilitates penetration across biological barriers and helps evade renal and hepatic clearance, thereby improving drug delivery efficiency and enabling controlled release [19]. In addition, nanocarriers promotes the accumulation of drugs at tumor sites, reduces off-target effects, and minimizes systemic toxicity, ultimately leading to superior therapeutic outcomes [20,21,22]. Nanomedicine platforms can be engineered for the co-delivery of immunomodulators, effectively converting immunologically “cold” tumors into responsive “hot” ones [23,24,25]. Such strategies can provoke robust and durable anti-tumor immune responses, demonstrating great potential in suppressing metastasis and preventing recurrence.

Metal ions play diverse and crucial roles in living organisms, particularly in immune functions [26]. They help maintain the normal functional state of the immune system by regulating immune cell activities, participating in inflammatory responses, and contributing to antioxidant defense [27,28,29,30,31,32]. Zinc is the second most abundant transition metal in the human body. As a signaling ion, free Zn^2+^ can modulate both innate and adaptive immune responses of immune cells, thereby influencing their functionality [33,34,35,36,37,38,39,40]. Zn^2+^ can induce tumor cell pyroptosis through both the Caspase-1/GSDMD-dependent canonical pathway and the Caspase-3/GSDME-dependent alternative pathway [41,42]. Zn^2+^ can directly enhance cGAS enzymatic activity and facilitate cGAMP synthesis, thereby efficiently priming and amplifying cGAS-STING signaling activation [43]. Meanwhile, excessive Zn^2+^ induces mitochondrial damage and intracellular ROS accumulation, which promotes the release of mitochondrial DNA into the cytosol; such leaked mitochondrial DNA acts as a key endogenous agonist to trigger and sustain cGAS-STING innate immune response [44]. Physiologically, the exonuclease TREX1 functions to degrade cytoplasmic DNA and maintain immune homeostasis, which restricts spontaneous cGAS-STING pathway activation [45]. Elevated Zn^2+^ interferes with this homeostatic mechanism, further promoting cytosolic DNA accumulation and potentiating cGAS-STING-mediated immune activation. Therefore, Zn-based nanomaterials are capable of stimulating ROS production in cancer cells, inducing immunogenic cell death (ICD), and ultimately enhancing anti-tumor immunity.

In recent years, cell membrane coating nanotechnology has increasingly emerged as a novel strategy for targeting specific cells and pathological tissues [46,47,48]. Owing to their abundant membrane receptors, favorable immune compatibility, and prolonged circulation time, cell membranes outperform many artificially synthesized surfaces [49,50]. This top-down biomimetic approach utilizes cell membrane-derived vesicles to camouflage nanoparticles (NPs) as natural materials, thereby endowing them with some inherent properties of their progenitor cells. For instance, when introduced into the systemic circulation, the coated membrane can reduce NP–bio interactions typically triggered by conventional synthetic surfaces [51]. Red blood cell membrane-coated NPs exhibit extended blood circulation time [46,52], platelet membrane-wrapped nanomaterials demonstrate selective adhesion to damaged blood vessels [53], and leukocyte membrane-coated silica microparticles possess the ability to traverse endothelial barriers [54]. Additionally, cancer cell membrane-camouflaged nanomaterials, which exhibit immune evasion and self-recognition capabilities, are becoming a promising targeting strategy in cancer therapeutics [55].

**Scheme 1:**
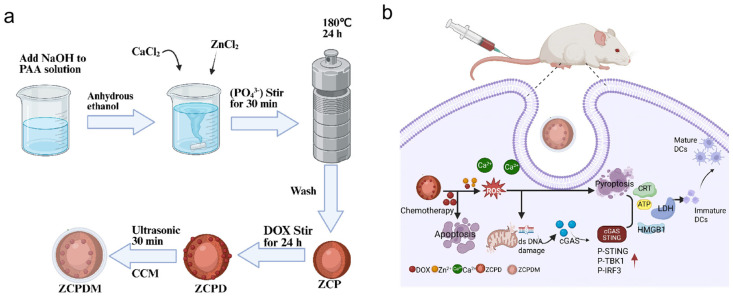
(**a**) Schematic representation of the synthetic strategy of ZCPDM drug delivery system. (**b**) Schematic diagram of ZCPDM inducing pyroptosis and amplifying cGAS-STING signaling for anticancer therapy.

Doxorubicin (DOX) is a potent clinical chemotherapeutic drug, but its clinical application is greatly limited by severe systemic and cardiac toxicity [56]. Nanocarrier-based delivery strategies have been proven effective to optimize DOX efficacy and reduce adverse effects [57]. Calcium phosphate (CaPs), the main component of bones, is known for its high biocompatibility and biodegradability, dissociating into Ca^2+^ and PO_4_^3−^ ions in acidic environments [58,59]. Herein, we designed and constructed a novel nanodrug delivery system (ZCPDM, Scheme 1a) by doping Zn^2+^ into a CaPs nanocarrier to form a Zn^2+^-doped CaPs carrier (ZCP), which was subsequently loaded with DOX. The surface of the carrier was further modified with homologous whole cancer cell membrane (CCM) to enable tumor cell-specific recognition and active uptake. Upon entering tumor cells, the CaPs degrades in response to the tumor-specific acidic microenvironment, releasing both DOX and Zn^2+^ (Scheme 1b). This process not only inhibits tumor cell proliferation but also induces intracellular ROS accumulation, leading to mitochondrial DNA (mtDNA) damage. The damaged mtDNA is sensed by cGAS, which in turn activates the downstream STING pathway and promotes immune responses. Furthermore, mitochondrial injury triggers the upregulation of inflammatory factors and activates the caspase-1/GSDMD signaling pathway, resulting in pyroptosis. This form of cell death releases additional mtDNA, creating a positive feedback loop that synergistically enhances STING pathway activity and amplifies antitumor immunity. The ZCPDM delivery system acts through multiple cooperative pathways to enhance tumor immunotherapy efficacy, significantly inhibiting tumor proliferation, recurrence, and metastasis. This work provides a new strategic approach for cancer treatment.

## 2. Materials and Methods

### 2.1. Synthesis of the ZCPDM Drug Delivery System

To synthesize uniform nanospheres with favorable morphology, different molar ratios of CaCl_2_ to ZnCl_2_ (1:9, 3:7, 5:5, 7:3, 9:1) were screened. Based on scanning electron microscopy (SEM) observations of their morphological characteristics, a CaCl_2_ to ZnCl_2_ ratio of 5:5 was ultimately selected as it yielded nanospheres with the best morphology and most uniform size. The specific synthesis steps are as follows:

Synthesis of ZCP: 72 mg of polyacrylic acid (PAA) was dissolved in 8.2 mL of deionized water under magnetic stirring. After complete dissolution, 1 mL of NaOH (1 M) was added. Subsequently, 40 mL of anhydrous ethanol was added, turning the solution milky white. While stirring continued, 0.25 mL of CaCl_2_ (1 M) and 0.25 mL of ZnCl_2_ (1 M) were added and allowed to react thoroughly. Subsequently, 0.3 mL of a 1 M phosphate buffer (Na_2_HPO_4_/NaH_2_PO_4_, 1:1 molar ratio) was introduced, and the mixture was stirred at room temperature for 20 min. The reaction was carried out hydrothermally at 180 °C for 24 h. After the reaction, the product was centrifuged at 11,000 rpm for 10 min. The supernatant was discarded, and the washing/centrifugation step was repeated three times. The collected precipitate was freeze-dried to obtain ZCP NPs.

Synthesis of ZCPD: 1 mg of DOX was dissolved in 1 mL of deionized water to prepare a 1 mg/mL solution. 2 mg of ZCP NPs were added to the DOX solution and magnetically stirred for 24 h. The mixture was then centrifuged at 11,000 rpm for 10 min. The supernatant was discarded, and the collected precipitate constituted the ZCPD sample.

Preparation of CCM: 4T1 cells were resuscitated and cultured until reaching 80–90% confluence. After washing with PBS, the cells were trypsinized, collected by centrifugation, and resuspended. Cell lysis was performed on ice using probe sonication (100 W, 60 cycles, with 3 s of sonication and 7 s of pause per cycle). The lysate was centrifuged at 4 °C and 1000 rpm for 10 min, and the supernatant was collected as the CCM.

Synthesis of ZCPDM: 1 mg of ZCPD was dispersed in 1 mL of CCM in PBS. The mixture was sonicated in an ice-water bath for 30 min and then centrifuged at 4 °C to collect the product, yielding the ZCPDM drug delivery system.

### 2.2. Determination of DOX Drug Loading Capacity and Encapsulation Efficiency in ZCPD

To evaluate the DOX loading capacity of ZCP, the supernatant was collected after centrifugation (11,000 rpm, 5 min) post-DOX loading. The UV absorption peak of DOX in the supernatant was measured using a UV spectrophotometer to confirm successful encapsulation. A standard curve was established by measuring the absorbance of a series of DOX standard solutions with gradient concentrations. The drug loading capacity (DLC) and encapsulation efficiency (EE) were calculated using the following formulas:

DLC (%) = (Mass of DOX fed − Mass of DOX in supernatant)/Mass of ZCP × 100%

EE (%) = (Mass of DOX fed − Mass of DOX in supernatant)/Mass of DOX fed × 100%

Given the inherent limitations of UV spectrophotometry for DOX quantification, including sensitivity and matrix interference, the presented values should be considered estimates.

### 2.3. Determination of CCM Encapsulation Efficiency in ZCPDM

SDS-PAGE gel electrophoresis and the Bradford protein assay were used to assess CCM encapsulation in ZCPDM. Samples of ZCP, ZCPD, ZCPDM, and CCM were lysed with protein lysis buffer for 30 min, mixed with SDS loading buffer, and heated in a boiling water bath for 5 min. The samples were then loaded onto a 10% SDS-PAGE gel. After electrophoresis, the gel was stained with Coomassie Blue and destained to visualize protein bands. Protein concentration was quantitatively determined using a Bradford assay kit.

### 2.4. Cell Culture

The 4T1 breast cancer cells is from Pricella Life Science & Technology Co., Ltd. (Wuhan, China). 4T1 cells were cultured in high-glucose DMEM medium supplemented with 10% fetal bovine serum and 1% penicillin/streptomycin at 37 °C in a 5% CO_2_ incubator. For cell resuscitation, frozen cells were quickly thawed, centrifuged, resuspended, and seeded. Passaging was performed when cells reached 80–90% confluence. Cells were trypsinized, centrifuged, resuspended, and subcultured into new flasks. For cryopreservation, cells were suspended in freezing medium (DMSO:FBS = 1:9), cooled gradually, and stored in liquid nitrogen.

### 2.5. Cellular Uptake

Prepared materials were co-stirred with Cy5-COOH dye for 24 h and then collected by centrifugation. 4T1 cells (1 × 10^4^) were seeded into confocal dishes. Dye-labeled materials were added to the dishes and incubated with the cells for 3 h and 6 h. After incubation, cells were washed with PBS, fixed, permeabilized with Triton X-100, stained with DAPI for nuclei, mounted, and observed under confocal laser scanning microscopy (CLSM). For flow cytometry analysis, 4T1 cells were seeded in 6-well plates at a density of 2 × 10^5^ cells/well and treated with 100 μg/mL of ZCPD or ZCPDM for 3 h and 6 h. After treatment, cells were trypsinized, collected, transferred to flow cytometry tubes, and analyzed using a Novo Express flow cytometer (version 2.2.0). Data were recorded and processed.

### 2.6. Mitochondrial Membrane Potential Assay

Changes in mitochondrial membrane potential were detected using a JC-1 assay kit. 4T1 cells were seeded in 6-well plates at a density of 1 × 10^5^ cells/well and incubated for 24 h. Cells were then treated with fresh medium (control), ZCP, ZCPD, or ZCPDM for 24 h. After treatment, the culture medium was removed, cells were washed with PBS, and 1 mL of fresh culture medium was added. 1 mL of JC-1 staining working solution was added, mixed thoroughly, and cells were incubated at 37 °C in the dark for 20 min. After washing, the red/green fluorescence intensity ratio was observed under a fluorescence microscope.

### 2.7. Intracellular ROS Detection

After co-incubating cells with materials for 12 h, DCFH-DA working solution (10 μM) was added and incubated for 30 min. Cells were washed with PBS, and green fluorescence was observed under a fluorescence microscope. For quantitative analysis, cells treated identically were trypsinized, collected, and intracellular ROS levels were measured by flow cytometry.

### 2.8. Validation of Tumor Cell Killing Ability

4T1 cells were seeded at a low density and allowed to grow to 80% confluence before material treatment. Cells were treated with different concentrations of materials (0, 12.5, 25, 50, 100, 200 μg/mL) for 24 h. CCK-8 working solution was then added, incubated for 1 h, and absorbance at 450 nm was measured to calculate cell viability. For live/dead staining, cells incubated with materials for 12 h were double-stained with FDA and PI, and observed under a fluorescence microscope (live cells: green; dead cells: red). Cell apoptosis levels were detected using an Annexin V-FITC Apoptosis Detection Kit (Beyotime, C1062, Shanghai, China) and analyzed by flow cytometry.

### 2.9. Cell Proliferation Activity Assay

After cell seeding, different treatment groups (PBS, ZCP, ZCPD, ZCPDM, all at a material concentration of 100 μg/mL) were added and cultured for 7–14 days. Culture was terminated when single clones contained ≥50 cells and were visible to the naked eye. The medium was removed, and cells were washed with PBS three times. Cells were fixed with paraformaldehyde and washed. Crystal violet staining solution was added to cover the cell surface, followed by staining at room temperature for 20–30 min. Plates were then rinsed repeatedly with PBS until the background was clear, air-dried, and imaged for colony counting. For CFSE-based proliferation assay, cells were labeled with CFSE, incubated for 24 h, collected by centrifugation, resuspended, and analyzed by flow cytometry.

### 2.10. Immunoactivation-Related Experiments

Cell morphology was observed by microscopy. Western Blot was used to detect Caspase-1 and GSDMD protein expression. Cell culture supernatants were collected, and LDH activity was measured according to the kit instructions. Immunofluorescence was used to observe the subcellular localization of HMGB1 and CRT. ATP levels in the supernatant were measured using an ATP detection kit. DNA damage was assessed via the comet assay and TUNEL staining. Pathway-related protein expression was detected by Western Blot. Levels of TNF-α, IL-6, and IFN-β in cell culture supernatants were measured by ELISA.

### 2.11. Animal Model Establishment

Female BALB/c mice aged 6–8 weeks were used. 4T1 cells (9 × 10^6^ cells in 100 μL PBS) were subcutaneously injected into the left lower inguinal region of the mice. Treatment began when the tumor volume reached approximately 50 mm^3^. Specifically, 6–8 week-old female BALB/c mice (purchased from Chongqing Ensiweier Biotechnology Co., Ltd., Chongqing, China) weighing 18–20 g were used for tumor model construction. Abdominal hair was removed with an electric shaver. A suspension of 9 × 10^6^ 4T1 cells in 100 μL PBS was injected subcutaneously into the left lower inguinal region. Tumor growth was monitored. Treatment commenced when the tumor volume reached about 50 mm^3^.

Mice were divided into 5 groups (*n* = 5 per group): PBS, ZCP, DOX, ZCPD, and ZCPDM. Treatment was administered via tail vein injection every 2 days for two weeks. Tumor volume and body weight were recorded every two days.

### 2.12. Evaluation of In Vivo Antitumor Efficacy

Seven days after tumor model establishment, when tumor volume reached 50 mm^3^, antitumor therapy was initiated in tumor-bearing BALB/c mice. Tumor-bearing mice were randomly divided into 5 groups (*n* = 5 per group) and received intravenous injections of: 200 μL PBS, 400 μL ZCP (2 mg/mL), 400 μL DOX (0.6 mg/mL), 400 μL ZCPD (2 mg/mL), or 400 μL ZCPDM (2 mg/mL). Injections were administered every two days for a total of 14 days. Mouse body weight and tumor volume were recorded during treatment. Tumor volume was calculated as: V_tumor = L × W^2^/2 (L: length, W: width). After treatment, mice were euthanized and dissected to collect solid tumors and other organs. Tumor tissues were placed in 4% paraformaldehyde for further sectioning and analysis.

### 2.13. Histological Analysis

Tumors and organs were fixed, paraffin-embedded, sectioned, and subjected to H&E staining, TUNEL staining, and immunohistochemical staining for HMGB1 and CRT. Stained sections were observed and photographed under an optical microscope.

### 2.14. Evaluation of Systemic Antitumor Immune Capacity

Lung tissues were perfused with India ink. A 15% India ink solution was injected into the lungs via the trachea to fill the entire lobes. The lungs were then washed with PBS and fixed overnight in Fekete’s solution. After fixation, lung metastatic nodules (white) against normal tissue (black) were observed.

### 2.15. Biosafety Evaluation

Hemolysis Assay: Red blood cells were co-incubated with materials for 1 h, centrifuged, and the absorbance of the supernatant at 540 nm was measured to calculate the hemolysis rate. Notably, DOX has inherent red color and characteristic absorption near 540 nm. To eliminate background interference from the material itself, equally concentrated material solutions were used to subtract background absorbance. The slight red supernatant observed in high-concentration groups was derived from released DOX rather than hemoglobin, confirming the negligible hemolysis rate.

Blood Indices and Tissue Sections: Blood was collected from treated mice for routine blood tests and biochemical analysis. The heart, liver, spleen, lungs, and kidneys were collected for H&E staining and pathological observation. Serum was collected to detect ALT, AST, CRE, and BUN levels to assess liver and kidney function.

### 2.16. Statistical Analysis

All quantitative data were presented as mean ± standard deviation (SD). Multiple group comparisons were performed using one-way ANOVA followed by Tukey’s post hoc test. Statistical significance was defined as * *p* < 0.05, ** *p* < 0.01, *** *p* < 0.001, and **** *p* < 0.0001. “ns” indicates not significant (*p* ≥ 0.05).

## 3. Results

### 3.1. Synthesis and Characterization of ZCPDM

**Figure 1:**
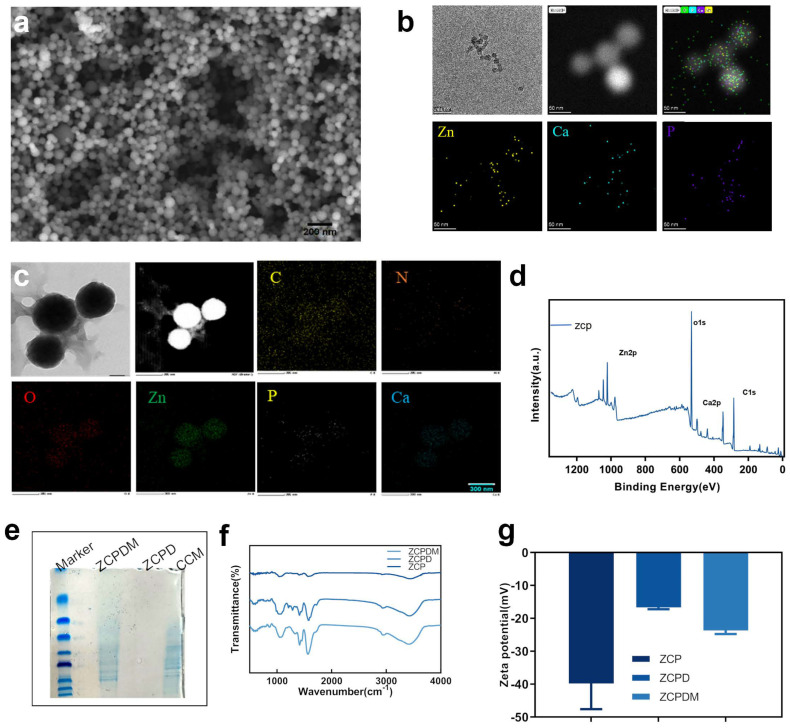
(**a**) SEM image of ZCP NPs. TEM image and elemental mapping of (**b**) ZCP and (**c**) ZCPDM NPs. (**d**) XPS spectrum analysis of ZCP NPs. (**e**) SDS-PAGE gel electrophoresis of ZCPDM, ZCPD and CCM. (**f**) FTIR spectra and (**g**) zeta potential analysis of ZCP, ZCPD and ZCPDM.

ZCP NPs were prepared *via* a hydrothermal reaction in a water-anhydrous ethanol system using PAA as a template. As shown in Figure S1, scanning electron microscopy (SEM) images of ZCP synthesized at varying CaCl_2_:ZnCl_2_ molar ratios reveal distinct morphological trends: spherical particles form when the ratio is below 5:5; at exactly 5:5, the ZCP NPs exhibit the smallest and most uniform size distribution (Figure 1a); and flocculent aggregates emerge when the ratio exceeds 5:5. Unified scale bars have been marked in all SEM images for intuitive and semi-quantitative comparison. Based on the calibrated scale bar, the 5:5 molar ratio group presents the most regular spherical morphology, narrowest particle size distribution and optimal monodispersity compared with other proportion groups. Given the optimal particle size and uniformity achieved at the 5:5, all subsequent characterization and validation experiments were conducted using ZCP NPs prepared at this molar ratio. The X-ray diffraction (XRD) pattern of ZCP NPs (Figure S2) showed no sharp crystalline diffraction peaks but rather a broad amorphous diffraction halo, indicating typical amorphous structural characteristics. Transmission electron microscopy (TEM, Figure 1b,c) images showed that both ZCP and ZCPDM NPs displayed a typical spherical morphology with good monodispersity and uniform size (Figure S3). The elemental mapping further confirmed the uniform distribution of O, P, Zn, and Ca elements within ZCP NPs, while C, N, O, P, Zn, and Ca elements were evenly distributed in ZCPDM NPs. The X-ray photoelectron spectroscopy (XPS) spectra clearly showed characteristic peaks of C 1s, O 1s, Zn 2p, and Ca 2p (Figure 1d). The DOX loading content in ZCPDM was quantified by UV-Vis spectroscopy (Figure S4) using a validated calibration curve, yielding a value of 30.4%. The DOX loading content in ZCPDM was quantified by UV-Vis spectroscopy (Figure S4) using a validated calibration curve, yielding a value of 30.4%.

To further confirm the successful coating of 4T1 whole cell protein (CCM) on the surface of the nanosystem, SDS-PAGE gel electrophoresis was performed for qualitative analysis of the protein composition. As shown in Figure 1e, the ZCPDM group displayed distinct bands at the corresponding protein molecular weight positions, while no protein bands were observed in the ZCPD group without CCM coating, indicating successful coating of 4T1-derived proteins onto ZCPD to form the ZCPDM nanosystem. Quantitative analysis of CCM encapsulation efficiency via the Bradford method showed that the encapsulation efficiency of CCM in ZCPDM was approximately 20%. The FTIR spectrum of ZCPDM NPs displayed characteristic peaks of anthraquinone ring skeleton vibration (1450–1500 cm^−1^) and stretching vibration of conjugated double bonds on the anthraquinone ring (1600–1650 cm^−1^), confirming successful loading of DOX onto ZCP (Figure 1f). Zeta potential measurements showed that the initial ZCP NPs had a surface potential of −39.8 mV, indicating high negative charge and good dispersion stability. Due to the positive charge of DOX molecules, after loading onto ZCP, the Zeta potential increased to −16.6 mV, confirming successful electrostatic adsorption of DOX onto ZCP. After coating with CCM, the Zeta potential decreased again to −23.7 mV (Figure 1g), further verifying effective CCM coating, which endowed the NPs with biomimetic camouflage properties and stability. The above results collectively confirm the successful construction of ZCPDM NPs.

### 3.2. ZCPDM Induces 4T1 Cell Death and Inhibits Proliferation

The tumor cell whole protein coating on the surface of ZCPD not only protects the drug during delivery but also enhances the targeting capability of the nano-delivery carrier toward cancer cells. First, by loading the nanomaterial with the Cy5 fluorescent dye, CLSM imaging and flow cytometry results showed that the red fluorescence signal intensity in the ZCPDM group was significantly higher than that in the ZCPD group (Figure S5). This confirms that CCM coating promotes cellular uptake of the material, and the difference between the 3 h and 6 h treatment groups indicates a positive correlation between the amount of material internalized and incubation time.

**Figure 2:**
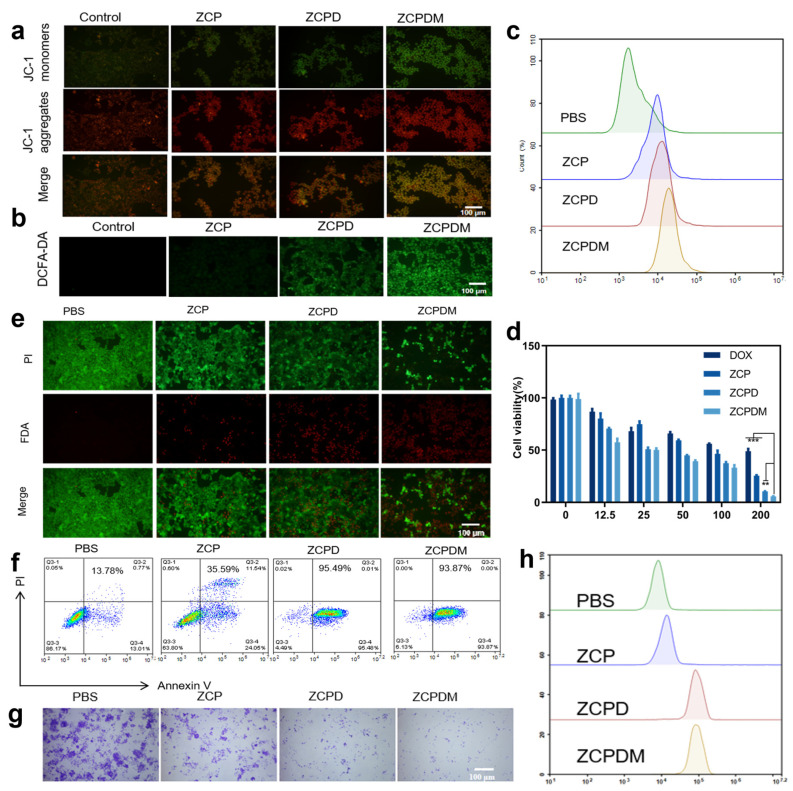
(**a**) Mitochondrial membrane potential of 4T1 cell after treatment with different materials. (**b**) ROS fluorescence images of 4T1 cell after treatment with different materials. (**c**) Flow cytometry analysis of intracellular ROS. (**d**) Cell viability of 4T1 cells after treatment with different concentrations of DOX, ZCP, ZCPD and ZCPDM for 24 h. (**e**) Live/dead cell levels after treatment with different materials. (**f**) Flow cytometry analysis of 4T1 cell apoptosis level after treatment with PBS, ZCP, ZCPD and ZCPDM for 24 h. (**g**) Cloning formation experiment for different material groups. (**h**) Flow cytometry analysis for the inhibition of proliferation of 4T1 cells after treatment with different nanomaterials.

Mitochondria are not only the primary sites for intracellular Zn^2+^ accumulation and utilization but also a major source of reactive oxygen species (ROS). Zn^2+^ entering mitochondria can induce mitochondrial DNA breaks, leading to elevated ROS levels and oxidative stress, thereby impairing mitochondrial function. To assess the impact of the ZCP series nano-drug delivery system on tumor cell mitochondrial function, the mitochondrial membrane potential of 4T1 cells treated with different materials was measured. JC-1 forms aggregates emitting red fluorescence under high membrane potential, while it exists as monomers emitting green fluorescence under low membrane potential. The results showed significantly enhanced green fluorescence and a marked decrease in the red/green fluorescence ratio in cells treated with ZCPD and ZCPDM, indicating a substantial drop in mitochondrial membrane potential (Figure 2a). This demonstrates that Zn^2+^ released from the NPs disrupts mitochondrial homeostasis, causing membrane depolarization, and further confirms the damaging effect of this drug delivery system on tumor cell mitochondrial function. Additionally, Zn^2+^ can induce rapid accumulation of endogenous ROS by inhibiting the mitochondrial electron transport chain. Therefore, the DCFH-DA fluorescent probe was used for ROS fluorescence imaging of 4T1 cells treated with different materials. DCFH-DA itself is non-fluorescent and can cross the cell membrane into the cytoplasm, where it is hydrolyzed by intracellular esterases to DCFH. In the presence of ROS, non-fluorescent DCFH is oxidized to fluorescent DCF. As shown in Figure 2b, cells treated with ZCPD and ZCPDM exhibited stronger green fluorescence, consistent with the flow cytometry results (Figure 2c). Thus, ZCPDM generates more ROS and exerts the strongest damaging effect on 4T1 cells, indicating significant therapeutic potential against tumors.

Next, the inhibitory and cytotoxic effects of the drug delivery system on 4T1 cells were evaluated. Cell viability was measured using a CCK-8 assay kit. As shown in Figure 2d, ZCPDM significantly inhibited 4T1 cell growth. Cell viability decreased progressively with increasing nanomaterial concentration. At a concentration of 100 μg/mL, the inhibitory effect of ZCPDM was markedly stronger than that of ZCPD, reducing cell viability to 33.23%. At 200 μg/mL, cell viability dropped to only 5.7%. To verify the involvement of specific pathways, rescue experiments were performed with the pyroptosis inhibitor MCC950 and the STING pathway inhibitor H-151 (Figure S6). Both inhibitors significantly restored cell viability, confirming that ZCPDM-induced cytotoxicity specifically relies on pyroptosis and STING pathway activation. These results indicate that this drug delivery system has significant proliferative inhibitory and cytotoxic effects on 4T1 cells. Furthermore, live/dead cell staining results (Figure 2e) showed a clear increase in dead cells after 6 h of treatment with ZCPDM, confirming its superior cytotoxic effect on 4T1 cells. Apoptosis analysis by flow cytometry revealed an apoptosis rate of 93.87% in the ZCPDM group, far exceeding the 13.9% in the PBS group (Figure 2f), demonstrating that the ZCPDM nano-delivery carrier effectively promotes tumor cell apoptosis with significant therapeutic efficacy.

Subsequently, a colony formation assay was conducted to evaluate the effect of the nano-delivery system on the proliferative capacity of 4T1 tumor cells. As shown in Figure 2g, the control (PBS) group formed a large number of colonies, while the ZCPDM treatment group showed a significant reduction in colony numbers, which was notably lower than other treatment groups such as ZCPD, ZCP, and DOX. This indicates that the nano-delivery system significantly inhibits the proliferative capacity of 4T1 tumor cells. To further verify the inhibitory effect of the ZCPDM nanoplatform on tumor cell proliferation, based on the colony formation assay, the CFDA SE cell proliferation tracking kit was used in combination with flow cytometry to quantitatively analyze the proliferation level of 4T1 cells. As shown in Figure 2h, the fluorescence intensity distribution peak of the ZCPDM treatment group shifted markedly to the right, with a slower decay rate of fluorescence intensity, indicating reduced cell division and significantly inhibited proliferative capacity. Compared to other treatment groups such as ZCPD and DOX, ZCPDM demonstrated a more pronounced effect in suppressing proliferation.

### 3.3. The Antitumor Mechanism of ZCPDM

**Figure 3:**
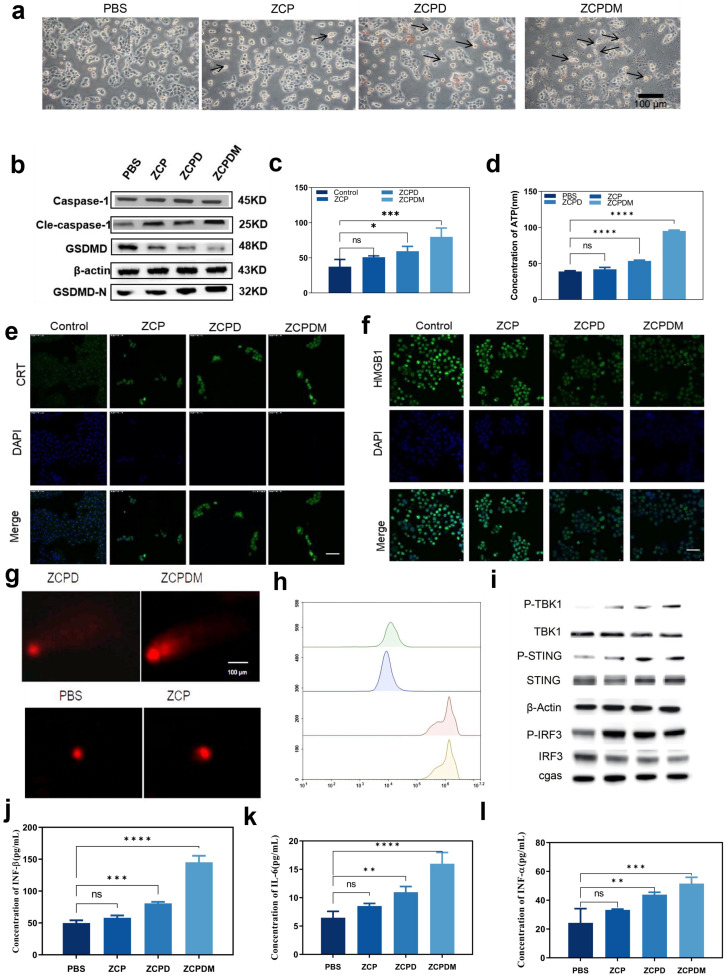
(**a**) Pyroptosis topography of different material groups. (**b**) Western blot analysis of Pyroptosis-related proteins. (**c**) LDH levels of 4T1 cells after treatment with PBS, ZCP, ZCPD, and ZCPDM for 24 h. (**d**) Quantitative analysis of ATP levels. Immunofluorescence staining images of (**e**) CRT and (**f**) HMGB1. (**g**) Comet assay for the assessment of DNA damage. (**h**) Flow cytometry analysis of TUNEL staining. (**i**) Western blot analysis of phosphorylation level cGAS-STING-associated proteins. ELISA results for antitumor cytokines (**j**) IFN-β, (**k**) IL-6 and (**l**) TNF-α.

When cells undergo pyroptosis, they typically exhibit swelling and enlargement. Before membrane rupture, numerous bubble-like protrusions, known as pyroptotic bodies, appear on the cell surface, followed by the formation of pores that compromise membrane integrity. This leads to the release of intracellular contents into the extracellular environment, thereby inducing an inflammatory response. As shown in Figure 3a, cells treated with ZCPDM displayed distinct protrusions. To further verify whether the ZCPDM nanosystem can induce pyroptosis in 4T1 tumor cells, the expression levels of key pyroptosis-related proteins—Cleaved Caspase-1, GSDMD, and its cleavage product GSDMD-N—were detected by Western blot. As shown in Figure 3b, the expression of Cleaved Caspase-1 (Cle-caspase-1) was up-regulated in the ZCPDM-treated group, indicating its conversion from the proenzyme form into the active state. Meanwhile, total GSDMD expression slightly decreased, while the cleavage product GSDMD-N was significantly up-regulated. These results suggest that activation of Cle-caspase-1 induces GSDMD cleavage, exposing its N-terminal fragment, which forms pores in the cell membrane and triggers pyroptosis. LDH release assay results showed that the ZCPDM group released the highest amount of LDH (Figure 3c), indicating the most severe membrane damage and cellular injury.

During non-apoptotic cell death such as pyroptosis, cells often undergo immunogenic cell death (ICD), releasing or exposing a series of damage-associated molecular patterns (DAMPs), including calreticulin (CRT), high mobility group box 1 (HMGB1), and extracellular ATP. These DAMPs can be recognized by immune cells such as dendritic cells, thereby initiating an anti-tumor immune response. To assess whether the ZCPDM nanosystem can induce ICD, three classic DAMPs—CRT exposure, HMGB1 translocation, and ATP release—were detected via immunofluorescence staining (Figure 3d–f and Figure S7). Results showed that ATP concentration released from the ZCPDM group significantly increased from 39 μM to 95 μM, further confirming the induction of ICD. The CRT signal intensity was notably enhanced in the ZCPDM group, indicating effective CRT translocation and ICD activation. In addition, compared with the PBS control, HMGB1 in ZCPDM-treated cells clearly translocated from the nucleus to the cytoplasm, suggesting nuclear leakage, which is a characteristic feature of ICD.

Next, intracellular DNA damage was assessed using the comet assay to evaluate the extent of DNA strand breaks. As shown in Figure 3g, cells treated with PBS showed no comet-like tails, whereas the ZCPD and ZCPDM groups displayed increased DNA fragmentation with long comet tails under electrophoresis, demonstrating that ZCPDM induced more severe DNA damage. Furthermore, TUNEL staining combined with flow cytometry showed increased mean fluorescence intensity in the ZCPD and ZCPDM groups (Figure 3h), indicating serious intracellular DNA damage. Elevated levels of DNA fragments can activate the cGAS-STING pathway. To confirm that the ZCPDM nanotherapeutic platform effectively induces activation of the cGAS-STING pathway, phosphorylation levels of key proteins in this pathway were examined by Western blot. As shown in Figure 3i, compared with other groups, the ZCPDM-treated group exhibited higher phosphorylation levels of TBK1, IRF3, and STING, confirming effective activation of the cGAS-STING immune pathway. Activation of this pathway induces secretion of IFN-β and proinflammatory cytokines, thereby initiating adaptive immunity and enhancing cancer immunotherapy. The secretion level of the chemokine IFN-β was measured by ELISA (Figure 3j), which showed that the ZCPD group significantly increased IFN-β expression, further demonstrating effective activation of the cGAS-STING pathway. The expression levels of other anti-tumor related cytokines, such as IL-6 and TNF-α, were also evaluated. Results indicated that the ZCPDM group exhibited a stronger effect in promoting the secretion of anti-tumor factors compared to other treatment groups.

### 3.4. Antitumor Efficiency in Mice Bearing 4T1 Tumors Treated with ZCPDM

**Figure 4:**
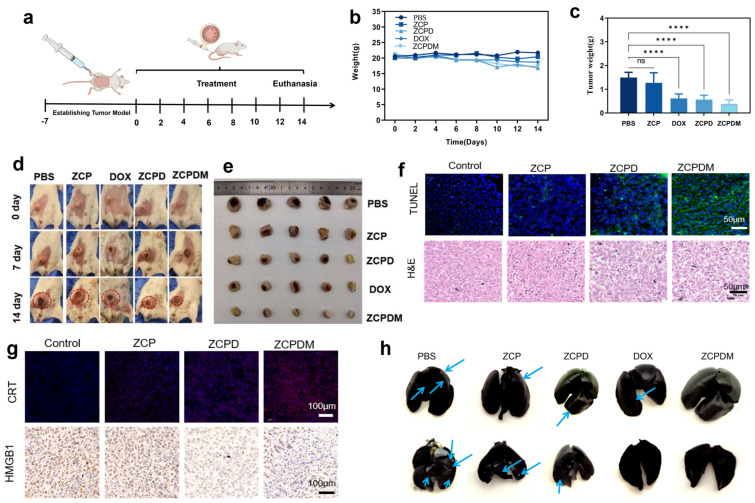
(**a**) Schematics depicting the therapeutic schedule in subcutaneous 4T1 cancer with intravenous injection of PBS, ZCP, ZCPD, and ZCPDM. (**b**) Curve of changes in the volume of solid tumors in mice. (**c**) Tumor tissue weight upon dissection. (**d**) Images of solid tumor growth in mice at different time points. (**e**) Photographic record of dissected tumor tissues. (**f**) H&E and TUNEL staining of mouse tumor tissues after treatment with different materials. (**g**) CRT and HMGB1staining of mouse tumor tissues after treatment with different materials. (**h**) India ink staining results of a single lung lobe in a mouse with implanted tumor model.

Next, a mouse tumor model of 4T1 cell-derived breast cancer was established to evaluate the in vivo antitumor efficacy of the materials (Figure 4a). PBS, ZCP, ZCPD, and ZCPDM were administered intravenously via tail vein injection into tumor-bearing mice, and changes in body weight and tumor volume were monitored. During the treatment period, mouse body weight showed no significant changes (Figure S8), while clear differences in tumor volume and weight were observed among the groups (Figure 4b,c and Figure S9). In the control group, tumors grew rapidly over the 21-day treatment period, indicating that PBS had little inhibitory effect. In contrast, the ZCPD treatment group achieved a tumor inhibition rate of 82.8%, demonstrating effective suppression of tumor growth. Tumor growth was photographed during the treatment (Figure 4d), and after treatment completion, tumors were excised, photographed, and weighed (Figure 4e). The results were consistent with the plotted tumor growth curves, confirming that the ZCPDM treatment group effectively inhibited solid tumor growth. These findings indicate that the synergistic effect of DOX loading and CCM coating significantly enhances tumor suppression, which is closely associated with the ZCPDM-mediated chemo-immunotherapy and the targeting function of CCM.

To further verify the antitumor activity of the ZCPDM nanosystem within tumor tissues, TUNEL staining and H&E staining were performed on 4T1 tumor sections to assess its effects on cell apoptosis and tissue structure. As shown in Figure 4f, tumor tissues in the ZCPDM group showed significantly enhanced green fluorescence, with a markedly increased proportion of apoptotic cells compared to control groups such as ZCPD, DOX, and PBS, indicating a significant pro-apoptotic effect of ZCPDM at the tissue level. H&E staining results revealed that the ZCPDM treatment group exhibited significantly reduced tumor cell density and typical apoptotic features such as cytoplasmic vacuolation, corroborating the TUNEL findings. Furthermore, immunofluorescence staining of tumor sections showed the most prominent red CRT signal in the ZCPDM group (Figure 4g), indicating substantial exposure of calreticulin on the tumor cell membrane. Concurrently, immunohistochemical staining showed a gradual decrease in HMGB1 signal within the nucleus. To further systematically investigate the regulatory effect of ZCPDM on the tumor immune microenvironment, we supplemented Immunofluorescence staining of typical immune markers, including CD4, CD8, CD86, and CD206 (Figures S10 and S11). The staining results demonstrated that ZCPDM treatment effectively promoted the infiltration of CD4^+^ and CD8^+^ tumor-infiltrating T cells, upregulated the M1-type macrophage marker CD86, and downregulated the M2-type macrophage marker CD206. These phenotypic changes in immune cells effectively remodeled the immunosuppressive tumor microenvironment and boosted systemic anti-tumor immune responses. These tissue-level results further confirm that ZCPDM can effectively induce immunogenic cell death (ICD) in vivo.

Tumor metastasis is a critical marker of disease progression. Since the lungs are a common site of metastasis, examining pulmonary metastasis can accurately reflect the extent of tumor dissemination in mice. Lung metastatic nodules were observed after India ink staining (Figure 4h). The control group showed obvious lung metastasis, with visible white nodules on both the frontal and dorsal surfaces. In contrast, the ZCPDM treatment group exhibited the fewest metastatic nodules. Quantitative statistical analysis revealed significant differences between groups, indicating that after activating immune pathways, the ZCPDM treatment effectively inhibited the metastasis of cancer cells to the lungs. These results further support the combined chemo-immunotherapeutic potential of ZCPDM in antitumor therapy.

Finally, the biocompatibility of the ZCPD nanotherapeutic carrier was investigated via a hemolysis assay. As shown in Figure S12, the average hemolysis rate in the H_2_O-positive control group was nearly 100%, while the rates in the material groups were all below 5%, indicating that the materials did not cause erythrocyte rupture and possessed good biocompatibility. Subsequently, mice were intravenously injected with a therapeutic dose of ZCPDM, and blood samples were collected at different time points to assess liver function, kidney function, and routine blood parameters. The results showed that multiple hematological indices, as well as liver and kidney function, in the ZCPDM-treated group remained within normal physiological ranges without significant abnormal fluctuations (Figures S13 and S14). Analysis of H&E-stained images of the heart, liver, spleen, lungs, and kidneys (Figure S15) showed that lung tissue changes in the PBS group originated from spontaneous 4T1 tumor metastasis rather than toxic damage, while only mild cytoplasmic vacuolation was sporadically observed in the liver of ZCPDM group without obvious inflammatory infiltration or necrosis. No treatment-related pathological abnormalities in cellular or tissue morphology in mice treated with either PBS or ZCPDM, indicating that the ZCPDM nanotherapeutic platform did not cause obvious toxic side effects in major organs. The above results demonstrate that, at effective doses over a 14-day continuous treatment period, the ZCPDM nanosystem induced no significant hematological toxicity or adverse effects on liver and kidney function in mice, exhibiting favorable biocompatibility and in vivo application safety. Meanwhile, tumor tissues were harvested for ICP quantification of zinc content to compare the delivery of free Zn^2+^ and nanoparticle-loaded Zn^2+^, as well as the targeting effect of the CCM-0 coating. Relevant data are presented in Figure S16, verifying that ZCPDM enables efficient Zn^2+^ enrichment in tumors and the coating modification optimizes in vivo targeting.

## 4. Discussion and Conclusions

Despite the favorable therapeutic efficacy and biocompatibility validated in this work, several limitations remain to be addressed. First, although Zn^2+^-induced mitochondrial damage and subsequent cGAS-STING pathway activation has been verified, the precise upstream molecular regulatory mechanisms underlying this immune modulation require further in-depth investigation. Second, the in vivo biosafety assessment of ZCPDM was conducted within a 14-day experimental cycle, which is insufficient to elucidate its long-term biodistribution, metabolic profile, and chronic toxicological risks. Future research will focus on exploring the detailed regulatory mechanism of Zn^2+^-mediated anti-tumor immune activation and performing long-term in vivo biosafety evaluation, aiming to provide more systematic and solid evidence for the clinical translational application of ZCPDM nanoplatforms.

In summary, this study successfully constructed a zinc-doped calcium phosphate-based nano-delivery system, ZCPDM, for the co-delivery of the chemotherapeutic drug doxorubicin (DOX) and homologous tumor cell membrane proteins. This system enables tumor-targeted drug delivery and degrades in the acidic tumor microenvironment, releasing DOX, Zn^2+^, and Ca^2+^. DOX directly induces apoptosis and elevates reactive oxygen species levels, while Zn^2+^ further exacerbates mitochondrial damage and DNA fragmentation. The released DNA fragments, together with Zn^2+^, activate the cGAS-STING immune pathway and simultaneously trigger pyroptosis, thereby promoting immunogenic cell death and the release of associated damage-associated molecular patterns, which enhances the anti-tumor immune response. In vitro experiments confirmed the system’s effectiveness at inducing DNA damage, activating the cGAS-STING pathway, and initiating pyroptosis. In a 4T1 tumor-bearing mouse model, ZCPDM demonstrated significant tumor inhibition, reduced lung metastasis, suppressed tumor recurrence, and exhibited low systemic toxicity alongside favorable biosafety. Notably, the obvious pulmonary pathological lesions observed in the PBS group further validated the successful establishment of the 4T1 high-metastasis tumor model, which further highlights the superior anti-metastatic capability of ZCPDM. Future work will focus on further exploring the molecular regulatory mechanism of ZCPDM against tumor metastasis, so as to lay a solid experimental foundation for the clinical translation of such biomimetic nanoplatforms. In conclusion, through a chemotherapy–immunotherapy synergistic mechanism, ZCPDM provides a promising strategy for achieving safe and efficient combined tumor therapy.

## Data Availability

The raw data supporting the conclusions of this article will be made available by the authors on request.

## Supplementary Materials

The following supporting information can be downloaded at https://www.mdpi.com/article/10.3390/jfb17060308/s1. Figure S1. SEM images of CaCl2 and ZnCl2 at different composite ratios, Scale bar: 200 nm.; Figure S2. XRD pattern of nanoparticles; Figure S3. (a) UV absorption peak of DOX supernatant after drug loading and (b) standard curve of DOX. Figure S4. The hydration particle size of ZCP, ZCPD, and ZCPDM. Figure S5. (a) CLSM images of ZCPD and ZCPDM after co-incubation with 4T1 cells for 3 h, 6 h; cell nuclei (blue), nanocarriers loaded with Cy5 dye (red); Scale bar: 100 μm. (b) Flow cytometry analysis of ZCPD and ZCPDM uptake by 4T1 after 3 h and 6 h of co-incubation with 4T1 cells. Figure S6. Cell viability after 24 h treatment with 50 μg/mL ZCPDM and different rescue reagents (25 μM H-151, 100 μM MCC950). Figure S7. Flow cytometry analysis of CRT levels of 4T1 cells after treatment with PBS, ZCP, ZCPD, and ZCPDM. Figure S8. Curve of changes in body weight of mice during treatment. Figure S9. Trends in tumor volume changes among different groups of mice. Figure S10. Immunofluorescence images of CD4+ and CD8+ in tumor tissue. Scale bar: 50 μm. Figure S11. Immunofluorescence images of CD206+ and CD86+ in tumor tissue. Scale bar: 50 μm. Figure S12. In vitro hemolysis experiments with different nanotherapeutic carriers. Figure S13. The main blood routine indexes of mice after treatment were leukocytes. (a) Red blood cells (RBCs), (b) white blood cells (WBCs), (c) basophils (BASs), (d) monocytes (MONs), (e) mean corpuscular hemoglobin mass (MCH), (f) lymphocytes (LYMs), (g) neutrophil percentage (NEU), (h) hemoglobin concentration (HGB), and (i) mean platelet volume (MCV). Figure S14. The main liver function and kidney function indexes of mice in different groups after treatment: (a) urea (UREA); (b) creatinine (CREA); (c) Albumin (ALB); (d) alkaline phosphatase (ALP); (e) Alanine amino acid transferase (ALT); (f) Aspartate amino acid transferase (AST). Figure S15. H&E staining of major organs; scale bar: 100 μm. Figure S16. Tumor tissue Zn^2+^ content at 9 h post-injection in mice treated with free Zn^2+^, uncoated ZCPD and CCM-coated ZCPDM. Table S1. The PDI values of different materials.

## Abbreviations

The following abbreviations are used in this manuscript:

ZCP	zinc-doped calcium phosphate
CaPs	Calcium phosphate
NCaPs	Nano-calcium phosphate particles
CCM	cancer cell membrane
DAMPs	damage-associated molecular patterns
DOX	doxorubicin
ROS	reactive oxygen species
ICD	immunogenic cell death
CRT	calreticulin
HMGB1	high mobility group box 1
PAA	polyacrylic acid
CLSM	confocal laser scanning microscopy
SEM	scanning electron microscopy
XRD	X-ray diffraction
XPS	X-ray photoelectron spectroscopy
TEM	Transmission electron microscopy
