# Supplementary material for: Zinc-Doped Calcium Phosphate Nanoagonists Amplifies cGAS-STING Signaling for Boosting Pyroptosis-Induced Cancer Immunotherapy
Source: J Funct Biomater. 2026 Jun 22;17(6):308. doi: 10.3390/jfb17060308 (PMC13301173; doi:10.3390/jfb17060308)
Supplement: Supplementary file 1 [file jfb-17-00308-s001.zip › jfb-4277668-supplementary.pdf]

Supporting Information

Supporting Figures

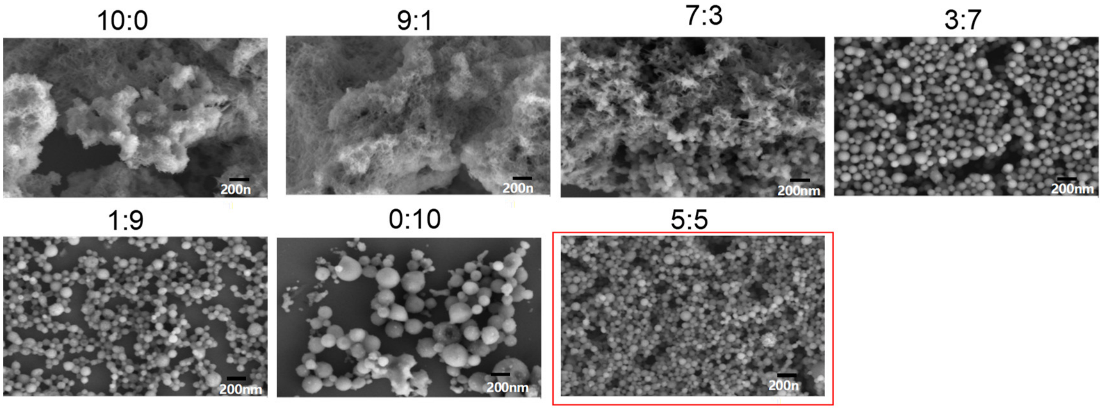

Figure S1. SEM images of $CaCl_2$ and $ZnCl_2$ at different composite ratios, Scale bar: 200 nm.

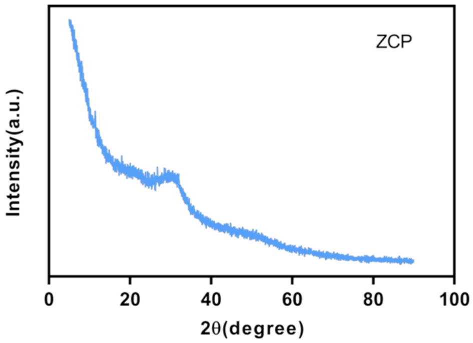

Figure S2. XRD pattern of nanoparticles.

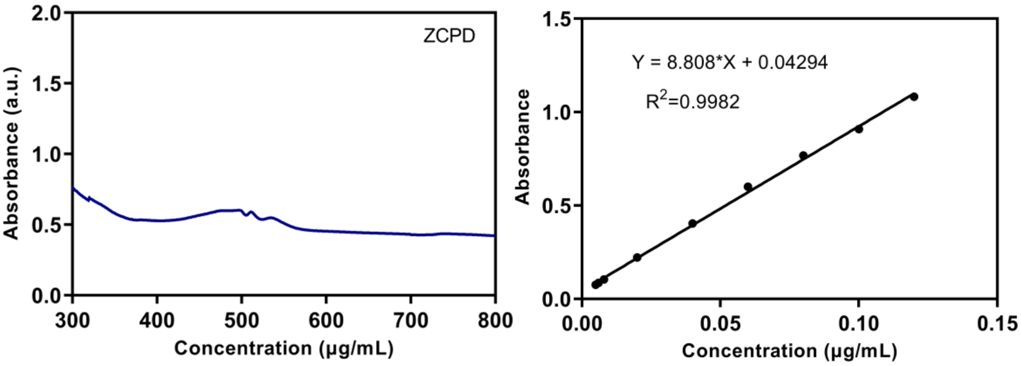

Figure S3. (a) UV absorption peak of DOX supernatant after drug loading and (b) standard curve of DOX.

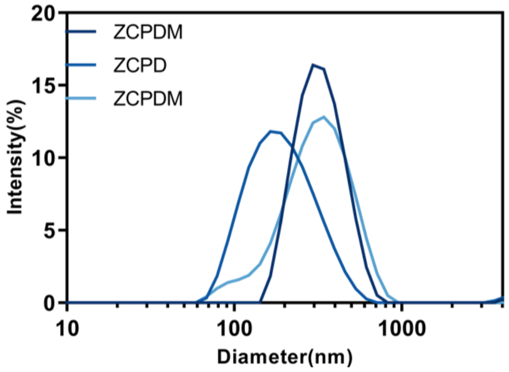

Figure S4. The hydration particle size of ZCP, ZCPD, and ZCPDM.

Table S1. The PDI values of different materials.

Sample	PDI
ZCP	0.173
ZCPD	0.252
ZCPDM	0.262

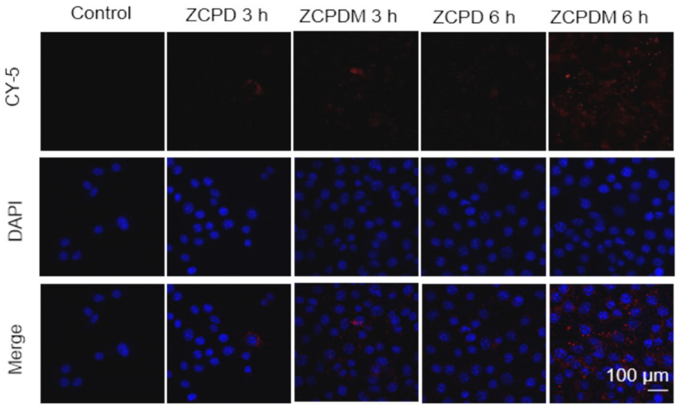

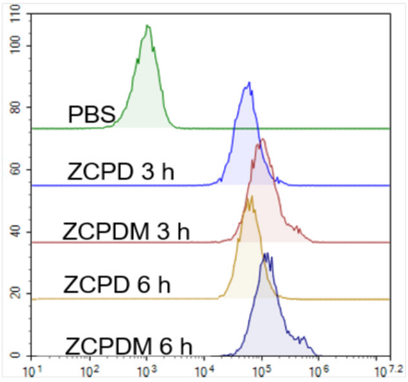

Figure S5. a) CLSM images of ZCPD and ZCPDM after co-incubation with 4T1 cells for 3 h, 6 h; cell nuclei (blue), nanocarriers loaded with Cy5 dye (red); Scale bar: 100 μm. b) Flow cytometry analysis of ZCPD and ZCPDM uptake by 4T1 after 3 h and 6 h of co-incubation with 4T1 cells.

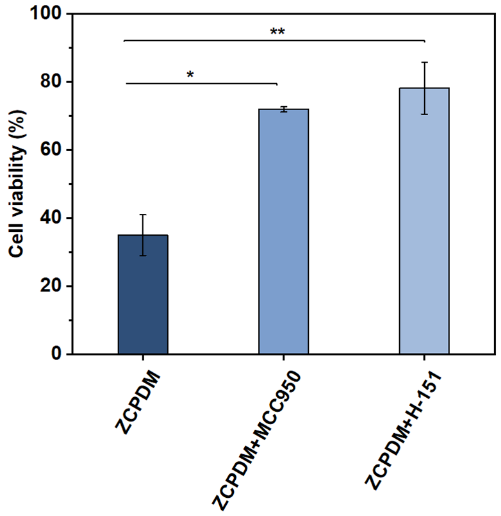

Figure S6. Cell viability after 24 h treatment with 50 μg/mL ZCPDM and different rescue reagents (25 μM H-151, 100 μM MCC950).

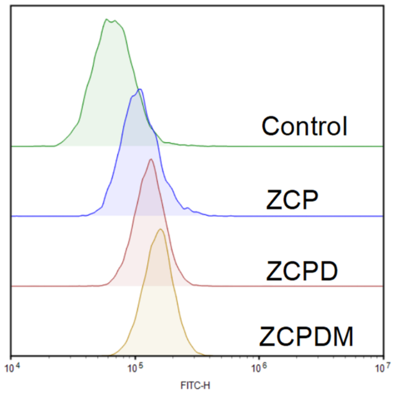

Figure S7. Flow cytometry analysis of CRT levels of 4T1 cells after treatment with PBS, ZCP, ZCPD, and ZCPDM.

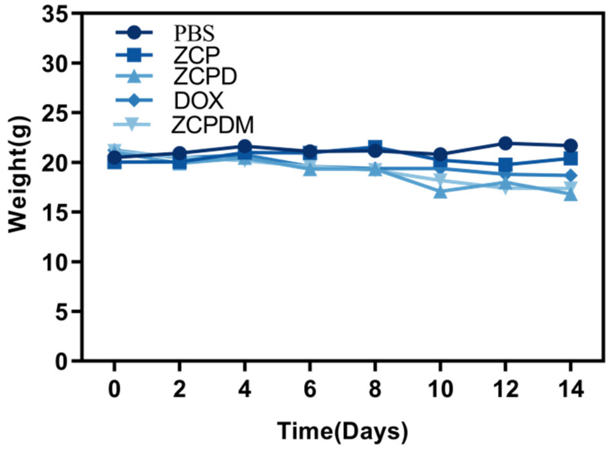

Figure S8. Curve of changes in body weight of mice during treatment.

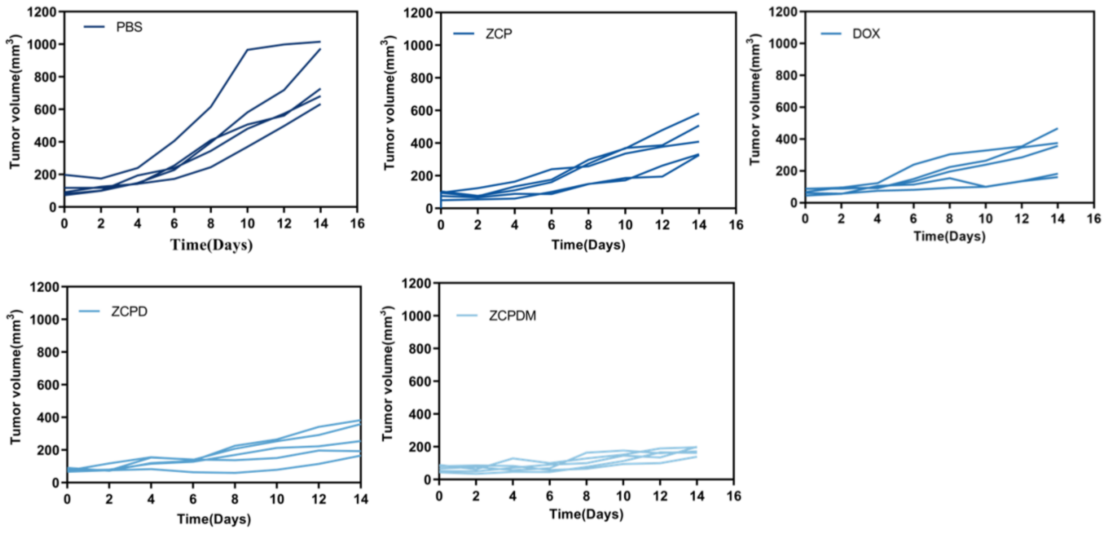

Figure S9. Trends in tumor volume changes among different groups of mice.

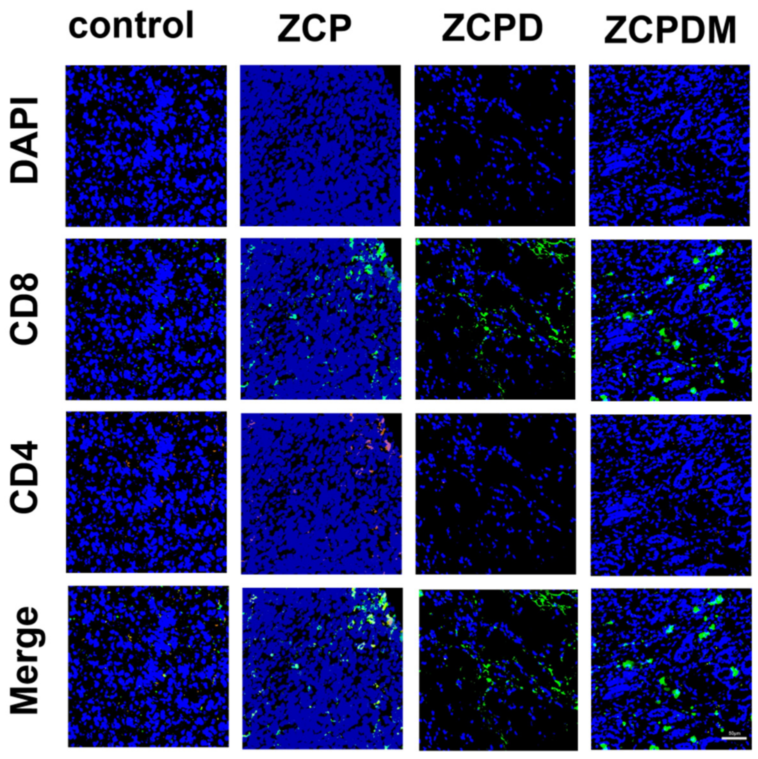

Figure S10. Immunofluorescence images of $CD4^+$ and $CD8^+$ in tumor tissue. Scale bar: 50 μm.

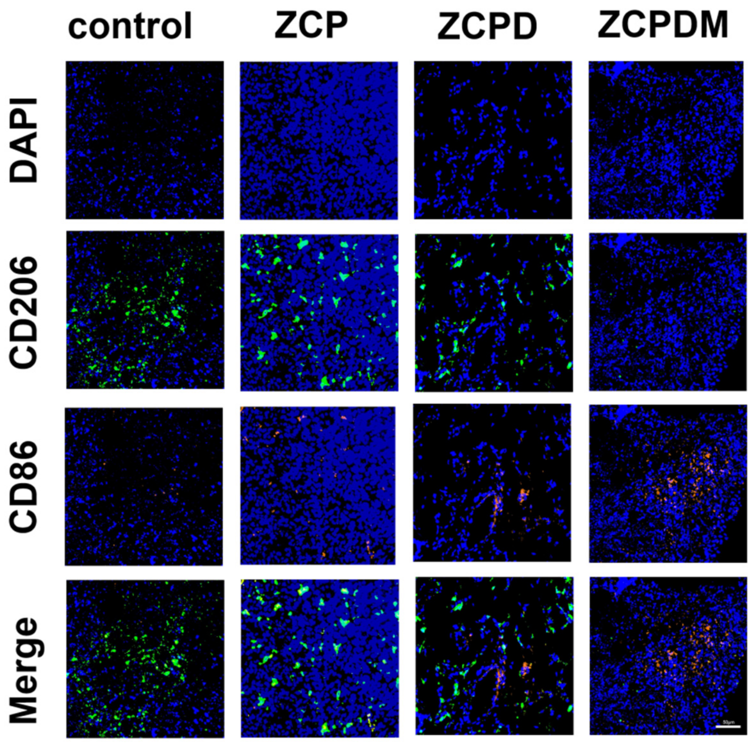

Figure S11. Immunofluorescence images of $CD206^{+}$ and $CD86^{+}$ in tumor tissue. Scale bar: 50 μm.

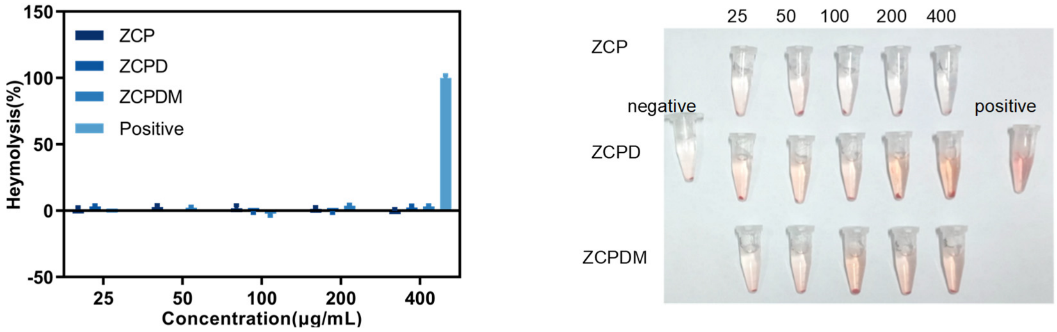

Figure S12. In vitro hemolysis experiments with different nanotherapeutic carriers.

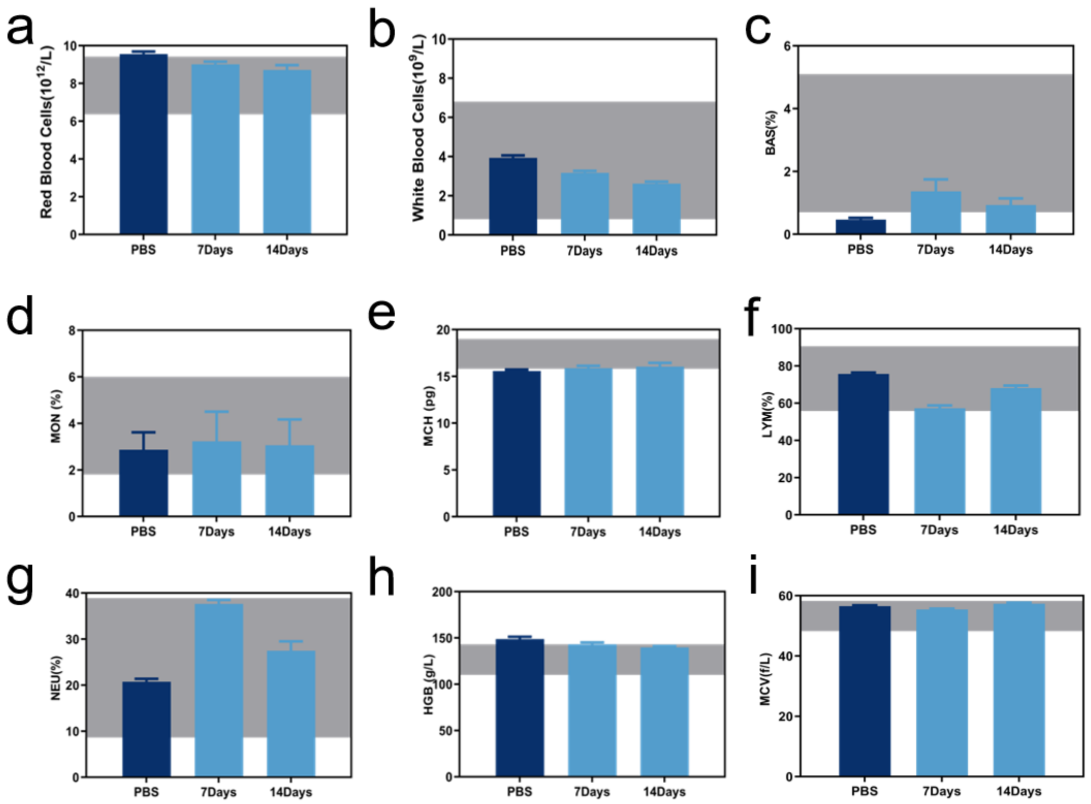

Figure S13. The main blood routine indexes of mice after treatment were leukocytes. (a) Red blood cells (RBCs), (b) white blood cells (WBCs), (c) basophils (BASs), (d) monocytes (MONs), (e) mean corpuscular hemoglobin mass (MCH), (f) lymphocytes (LYMs), (g) neutrophil percentage (NEU), (h) hemoglobin concentration (HGB), and (i) mean platelet volume (MCV).

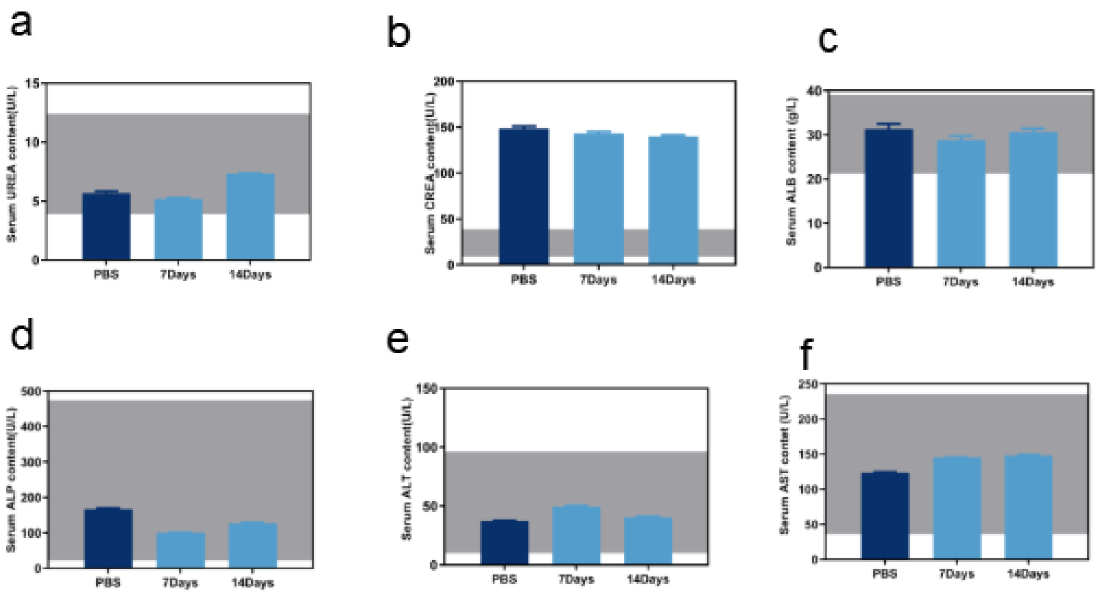

Figure S14. The main liver function and kidney function indexes of mice in different groups after treatment: (a) urea (UREA); (b) creatinine (CREA); (c) Albumin (ALB); (d) alkaline phosphatase (ALP); (e) Alanine amino acid transferase (ALT); (f) Aspartate amino acid transferase (AST).

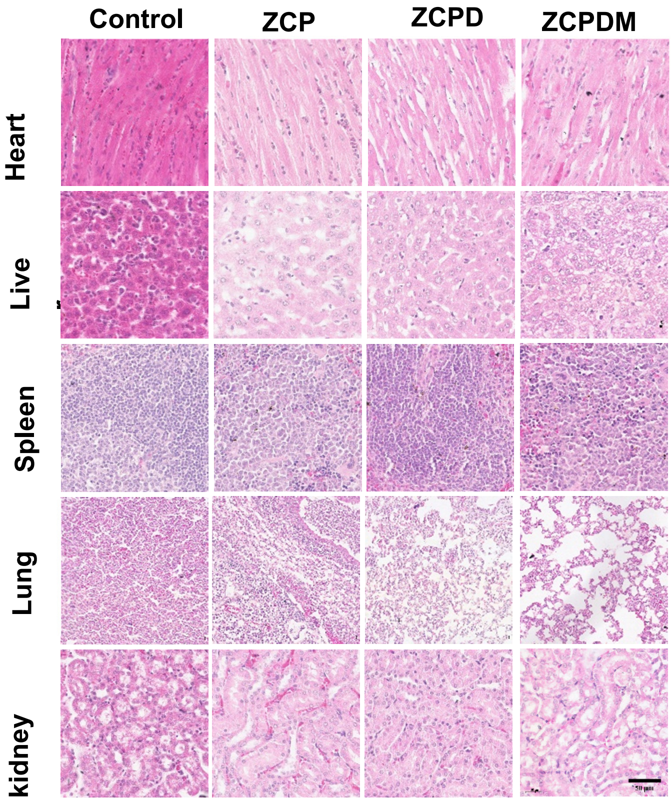

Figure S15. H&E staining of major organs; scale bar: 100 µm.

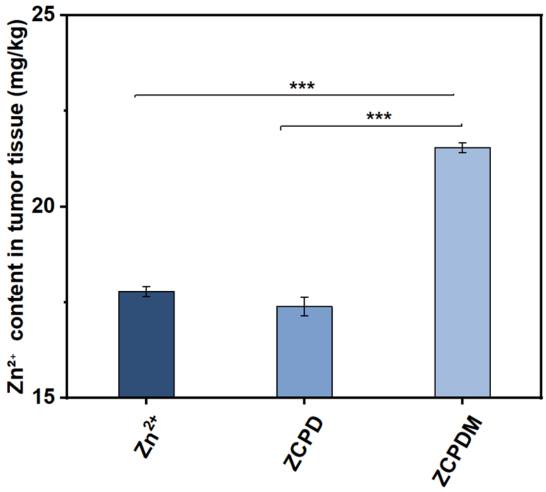

Figure S16. Tumor tissue Zn^{2+} content at 9 h post-injection in mice treated with free Zn^{2+}, uncoated ZCPD and CCM-coated ZCPDM.
